# Prevalence of Low Bone Mineral Density in a Low-Income Inner-City Population

**DOI:** 10.1002/jbmr.221

**Published:** 2010-08-18

**Authors:** Diala El-Maouche, Xiaoqiang Xu, Joseph Cofrancesco, Adrian S Dobs, Todd T Brown

**Affiliations:** Department of Medicine, Johns Hopkins University School of Medicine Baltimore, MD, USA

**Keywords:** OSTEOPOROSIS, BONE MINERAL DENSITY, HIV, BMI, INNER CITY

## Abstract

Bone mineral density (BMD) is an important factor linked to bone health. Little is known of the prevalence of low BMD and its associated risk factors in an urban underserved population. Between 2001 and 2004, we recruited 338 subjects who completed drug use and medical history questionnaires, underwent hormonal measurements, and underwent whole-body dual-energy X-ray absorptiometry (DXA) for evaluation of BMD and body composition. Of these, 132 subjects had site-specific DXA (lumbar spine and hip) performed. Osteoporosis was defined as a *T*-score of –2.5 or less for men 50 years of age and older and postmenopausal women and a *Z*-score of –2.0 or less in men younger than 50 years of age and premenopausal women at either the lumbar spine, total hip, or femoral neck, according to National Osteoporosis Foundation (NOF) guidelines. The cohort consisted of mostly African-American, middle-aged people with a high prevalence of illicit drug use, 50% HIV^+^, and 39% hepatitis C^+^. Osteoporosis was identified in 22% of subjects (24 men, 5 women), with the majority of cases (90%) attributable to osteoporosis at the lumbar spine. Osteoporosis was more common in men than in women. Lower whole-body BMD among women was associated with multiple risk factors, but only with lower lean mass among men. Osteoporosis was highly prevalent in men, mainly at the spine. The risk factors for bone loss in this population need to be further clarified. Screening men for osteoporosis starting at age 50 might be warranted in this population given the multiple risk factors and the unexpectedly high prevalence of low BMD. © 2011 American Society for Bone and Mineral Research.

## Introduction

Osteoporosis is a disease of the bone characterized by decreased bone mineral density (BMD) and bone quality with a resulting increased risk in skeletal fragility and fracture.(1) Fractures related to osteoporosis represent a large burden for society and high health care costs for prolonged hospitalization and complications of increased morbidity and mortality, as well as indirect costs of declining functioning, nursing home placement, and reduced quality of life.(2) Public health awareness of osteoporosis has lead to increased interest in identifying the fracture risk factors because fractures are preventable by screening appropriate populations and initiating effective therapy. Most of the data regarding osteoporosis has been derived from postmenopausal female white populations. Only recently is osteoporosis in men gaining attention(3,4) and, to a lesser extent, osteoporosis in African Americans.

Men and women living in the inner city have multiple risk factors for osteoporosis, but the prevalence of low BMD is not well defined in this population. Human immunodeficiency virus (HIV) infection, hepatitis C infection, alcohol abuse, smoking, illicit drug use, hypogonadism, homelessness, and poor nutritional status are common in inner-city populations(5–9) and have been associated with increased risk of osteoporosis and fractures.(10–13)

In this study, we aimed to determine the prevalence of osteoporosis and reduced BMD in a cohort of inner-city Baltimore's population and investigate the risk factors associated with BMD.

## Subjects and Methods

### Study population

The Study of HIV, Injection Drug Use, Nutrition and Endocrinology (SHINE) is a cross-sectional study of volunteers in Baltimore designed to evaluate the effects of HIV infection and drug use on multiple endocrine and metabolic parameters. Between 2001 and 2004, subjects were recruited from local medical and HIV clinics, community methadone maintenance programs, an existing cohort of injection drug users, homeless shelters, and by word of mouth. Subjects between 18 and 65 years of age were eligible for recruitment, which was stratified by gender and HIV status. Individuals with any of the following characteristics were excluded: (1) known gonadal dysfunction by medical history, (2) other significant medical problems resulting in a serum creatinine concentration more than three times the upper limit of normal (ULN), transaminases more than three times ULN, or hematocrit less than 25%, and (3) untreated endocrine problems, such as hypothyroidism, as indicated by medical history. All volunteers had provided written informed consent before participation. The study was approved by the Johns Hopkins University Institutional Review Board.

### Drug and alcohol use classification

Drug-use status was divided into three groups of (1) no use, (2) occasional (<3 times/week), and (3) heavy drug users (≥ 3 times/week) over the past 6 months. Drug use included heroin (injection, snorting, or smoking), cocaine (snorting or injection), speedball (injection), crack/freebase, marijuana, and street methadone. Those who were enrolled in a methadone treatment program for at least 3 months were grouped separately. Subjects also were stratified by alcohol use into three groups of (1) no use, (2) moderate (<3 drinks/day), and (3) heavy use (≥ 3 drinks/day) during the past 6 months. Drinking equivalents for one drink included 8 fluid ounces of beer or wine, 4 fluid ounces of liquor or malt, or 1 fluid ounce of vodka. Subjects who reported smoking in the past 6 months were considered smokers.

### HIV and hepatitis C infection

HIV status was determined by (1) self-reported serostatus, (2) self-report of antiretroviral therapy, or (3) ELISA testing followed by Western blot confirmation for subjects without documentation of HIV status. Hepatitis C status was self-reported.

### Biochemical measurements

Hormonal measurements included free testosterone (fT), follicle-stimulating hormone (FSH), luteinizing hormone (LH), dehydroepiandrosterone (DHEA), and estradiol from morning serum samples collected between 8:00 and 10:00 a.m. in the Clinical Trials Unit at the Johns Hopkins University School of Medicine. Free testosterone was measured by equilibrium dialysis (Esoterix, Inc., Austin, TX, USA) with a normal range of 52 to 280 ng/dL for men. Intraassay coefficients of variation (CV) ranged from 6.6% to 9.4%, and interassay CV ranged from 9.1% to 11.9%. Males were considered hypogonadal if they had an fT level of less than 52 ng/dL or were on testosterone-replacement therapy. Estradiol was measured by radioimmunoassay (Esoterix, Inc.) in which intra- and interassay CVs were 5.2% and 8.0%, respectively. This variable was divided into tertiles from lowest (1) to highest (3) when evaluating its relation to BMD. LH and FSH were measured by immunochemiluminometric assay (ICMA), for which the intra- and interassay CVs were 3.4% and 3.8%, respectively, for LH, and 3.2% and 6.7%, respectively, for FSH. Females were considered postmenopausal if they had a FSH concentration of less than 50 mIU/mL, age of 51 years of greater, or they self-reported when the FSH level was greater than 30 and 50 mIU/mL or less.

Using frozen serum samples, parathyroid hormone (PTH) and vitamin D levels were obtained later on 126 subjects of the subset with site-specific dual-energy X-ray absorptiometry (DXA) scans. Intact PTH (iPTH) was measured by two-site ELISA (Alpco Diagnostics, Salem, NH, USA) with intraassay CVs of 2.8% to 3.0% and interassay CVs of 5.1% to 5.5%. Reading the assay at 450 nm, iPTH concentrations were valid up to about 200 pg/mL as the upper limit. Secondary hyperparathyroidism was defined as iPTH greater than 65 pg/mL. 25-Hydroxyvitamin D [25(OH)D] level was measured by radioimmunoassay (DiaSorin, Stillwater, MN, USA) with an intraassay CV of 8.6% to 11.7% and interassay CV of 8.2% to 11.0%. The lower limit was measured at 1.5 ng/mL or less. Vitamin D deficiency was defined as 25(OH)D concentration of 15 ng/mL or less.

Measurement of serum inflammatory markers, including tumor necrosis factor α (TNF-α), high-sensitivity interleukin-6 (hs-IL6), and high-sensitivity C-reactive protein (hs-CRP), were completed at the Advanced Chemistry Laboratory at the Johns Hopkins University using an ALPO assay (Windham, NH, USA). TNF-α was measured by ELISA, where intra- and interassay CVs were 8.2% and 6.1%, respectively. hs-IL6 was measured by ELISA with intra- and interassay CVs of 5.1% and 5.0%, respectively. hs-CRP was measured by ELISA with intra- and interassay CVs of 6.3% and 2.2%, respectively. These markers were divided into tertiles from lowest (1) to the highest (3) level to evaluate the relation between BMD and inflammatory markers.

### Body composition and BMD measurements

Body mass index [BMI = weight (kg)/height^2^ (m^2^)] was calculated for all subjects

Whole-body DXA scans were performed on a Hologic 4500A machine with QDA4500A software Version 9.03 (Hologic Inc, Bedford, MA, USA) to obtain measures of total lean body mass (LBM, kg), fat body mass (kg), as well as whole-body BMD (WBMD, g/cm^2^). A subset of the participants had BMD assessed regionally at the lumbar spine and hip (total and femoral neck). For this subset of patients, *T*-scores and *Z*-scores were calculated from the site-specific BMD measures using normative data from the manufacturer matched for gender and race.(14,15) Subjects who specified race other than African American, white, or Hispanic (*n* = 5) were considered white for consistency of definition (non-African American) because data are available only for whites, African Americans, and Hispanics. Osteoporosis was defined as a *T*-score of –2.5 or less for men 50 years of age or older and postmenopausal women and a *Z*-score of –2.0 or less in men younger than 50 years of age and premenopausal women at either the lumbar spine, total hip, or femoral neck, according to National Osteoporosis Foundation (NOF) guidelines.(16) Reduced BMD was defined as a *T*-score of less than –1.0. Osteopenia was defined as –2.5 < *T*-score < –1.

### Statistical analysis

Data were stratified by gender, and the demographic characteristics and BMD measures of the study participants were described. Normality of the distributions for each variable was checked by plotting histograms. The first part of the analysis included the larger cohort (total study population) with WBMD (continuous variable) as the dependent variable in order to obtain more power and perform correlative analysis stratified by gender. WBMD closely reflects assessment by site-specific measures and has an excellent correlation with spine BMD in predicting osteoporosis.(17) The analysis examined the relationship between WBMD and various independent variables (risk factors) and was stratified by gender. One-way ANOVA was used to determine the association between WBMD and categorical independent variables, and linear regression analysis was used to assess the relation between WBMD and continuous independent variables. The association between WBMD and multiple variables was done by stepwise backward regression analysis using a *p* value of .25 to enter and .1 to remain in the model. Variables tested included age, gender, race (African American or other), smoking status (yes/no), alcohol/drug use (none, moderate, or heavy), methadone program (yes/no), hypogonadal/menopausal, hepatitis C status, HIV status, BMI or LBM + fat mass, and lab parameters (ie, DHEA, estradiol, TNF-α, IL6, and CRP) in tertiles. The final model locked in age and race and was stratified by gender because these variables are known risk factors for low BMD.

The second part of the analysis investigated the subset of participants with site-specific DXA, where osteoporosis (as defined earlier) was considered the dependent variable (dichotomous). A chi-square test was used to determine the group differences of osteoporosis among categorical independent variables. Regression analysis was used to determine the relation between osteoporosis and continuous independent variables. Multivariate backward stepwise regression was performed similar to the WBMD model described earlier. Smaller sample size limited stratification by gender, but the final model locked in gender, along with age and race. Risk factors tested were the same as used for WBMD analysis. In addition, we evaluated the relationship between osteoporosis and vitamin D deficiency status [25(OH)D ≤ 15 ng/mL) and the presence of secondary hyperparathyroidism (iPTH > 65 pg/mL).

*p* Values less than .05 were considered significant. Statistical analysis was performed on JMP Statistical Software 7.0.2 (2007, SAS Institute, Inc., Cary, NC, USA).

## Results

### Demographic characteristics of study participants

The cohort consisted of a total of 338 subjects who had measures of WBMD, including 187 males and 151 females. Demographic characteristics stratified by gender are presented in Table 1, with similar distributions among women and men. The population consisted of a relatively young group (mean age 42.8 years) of mostly African-American (93.8%) inner-city men and women of low socioeconomic status (SES); 27% of the population was homeless in the prior year, 15% were employed at the time of the study, 51% reported income of $10,000 or less in the prior year, and 28% reported no legal income. Seventy percent reported moderate or heavy drug use, and 11% reported the consumption of three or more alcohol drinks per day. Most were smokers (84%). Ninety-nine subjects (30%) were enrolled in a methadone program, of which 66 also were active drug users. Half (50%) the population was HIV^+^, and 38.7% were hepatitis C^+^. The prevalence of hypogonadism was 25% in men and 21% in women (postmenopausal). One subject was on testosterone-replacement therapy (fT = 2.2 ng/dL, therefore hypogonadal by biochemical definition), one subject reported corticosteroid use; and three subjects were taking anticonvulsant medications. Men had a statistically significant lower BMI than women (24.8 and 27.4 kg/m^2^, respectively, *p* < .05) as well as lower fat mass but higher LBM and WBMD (Table 1).

**Table 1:** Population Demographics and Biochemical Measures

	WBMD cohort (*n* = 338)		Site-specific DXA cohort (*n* = 132)	
			
Variable	M (187)	F (151)		
Age (mean, SD)	43.3 (7.2)	42.1 (7.6)	42.5 (8)	
Male	—	—	83 (63%)	
African American	175 (94%)	141 (93%)	124 (94%)	
Homeless (in the past year)	48 (26%)	41 (28%)	35 (27%)	
Employed (at time of study)	32 (17%)	19 (13%)	12 (9%)	
High school or GED completion	114 (62%)	77 (51%)	72 (55%)	
Smoking^a^	145 (82%)	128 (87%)	102 (80%)	
Alcohol^b^				
No use	74 (41%)	73 (50%)	55 (43%)	
<3 drinks/day	85 (48%)	58 (39%)	57 (45%)	
≥3 drinks/day	20 (11%)	16 (11%)	16 (13%)	
Methadone^c^	43 (24%)	56 (37%)	33 (25%)	
Drug use^d^				
No use	49 (28%)	47 (33%)	23 (18%)	
<3 times/week	74 (42%)	44 (31%)	47 (37%)	
≥3 times/week	54 (30%)	53 (37%)	58 (45%)	
Hepatitis C^e^	71 (39%)	58 (40%)	49 (38%)	
HIV^f^	103 (56%)	65 (43%)	67 (51%)	
Hypogonadal^g^/menopausal^h^	44 (25%)	32 (21%)	36 (28%)	
BMI (kg/m2) (mean, SD)	**24.8 (4.4)**	**27.4 (6.6)**	25.1 (5)	
Lean body mass (kg) (mean, SD)	**59.3 (7.9)**	**47.2 (8.1)**	54.4 (9.8)	
Fat body mass (kg) (mean, SD)	**13.4 (7.4)**	**22.3 (11)**	15.8 (8.9)	
25 (OH) D (ng/ml) (mean, SD)	—	—	16.1 (0.65)	
25 (OH)D< 15	—	—	61 (48%)	
iPTH (pg/ml) (mean, SD)	—	—	**51.3 (2.2)**	
iPTH> 65	—	—	**28 (22%)**	
TNF-α (pg/ml) (mean, SE) N = 243	2.9 (0.26)	2.6 (0.3)	2.8 (0.3)	
hsCRP (ng/ml) (mean, SE) N = 243	3.9 (0.6)	5.1 (0.9)	6.3 (1.1)	
hsIL-6 (pg/ml) (mean, SE) N= 238	3.3 (0.23)	3.8 (0.3)	3 (0.2)	
			**M**	**F**
Free T (ng/dL) (mean, SE) N = 318	**89.1 (4.9)**	**5.5 (1.4)**	**83.4 (6.8)**	**2.6 (0.3)**
Estradiol (pg/ml) (mean, SE) N = 327	**32.7 (1.5)**	**52.5 (4.5)**	**37.1 (2.2)**	**52.4 (6.4)**
LH (mIU/ml) (mean, SE) N = 328	**5.6 (0.26)**	**12 (1.2)**	**5.7 (0.4)**	**12.9 (2.4)**
FSH (mIU/ml) (mean, SE) N = 328	**5.8 (0.5)**	**15.9 (1.7)**	**5.9 (0.8)**	**19.3 (3.5)**
DHEA (ng/dL) (mean, SE) N = 328	**161 (8.5)**	**107 (7.8)**	**178 (15)**	**119 (15)**

*Note:* Items in **boldface** denote statistically significant difference (*p* < .05) between males and females.

a Determined by answering yes to having smoked cigarettes in the past 6 months (irrespective of quantity).

b Equivalent of 1 drink is 8 fluid ounces of beer or wine, 4 fluid ounces of liquor or malt, and 1 fluid ounce of vodka.

c Includes subjects who are enrolled in a methadone treatment program for at least 3 months.

d Includes marijuana, crack/freebase, cocaine (snorting, injection), speedball (injection), heroin (smoking, snorting, injection), and street methadone in the past 6 months.

f Self-report.

g Self-report, HIV-positive by testing, or history of past or present use of antiretroviral therapy.

h Defined as having a free testosterone level < 52 pg/mL or currently on testosterone-replacement therapy.

i Defined as having FSH level > 50 mIU/mL, age ≥ 51 years, or FSH level > 30 mIU/mL and ≤ 50 mIU/mL and answered yes to having gone through menopause.

### Factors associated with WBMD

When stratified by gender, the relation of WBMD to various independent risk factors showed major differences among men and women (Table 2). Whereas only LBM was positively related with BMD in men, multiple risk factors showed significant associations in women. In females, older age, non-African American race, postmenopausal status, lower LBM, and methadone use showed significant association with lower BMD in univariate analysis. Similar results were obtained when BMI replaced fat mass and LBM, with BMI as a significant determinant. Methadone use was an independent determinant of WBMD in women, where the association was not affected by simultaneous adjustment for other factors. Moderate (<3 drinks/day) consumption of alcohol was significantly associated with higher BMD in women. Women having the highest levels of DHEA and estradiol had significantly higher BMD measures, consistent with the known benefits of estrogen on bone. Independent determinants of lower BMD in women included increasing age, non-African-American race, methadone, and lower LBM (Table 3). There was a trend toward lower BMD by HIV status in women (*p* = .11). Among HIV-infected women (*n* = 65), we explored whether the self-report of antiretroviral therapy was associated with WBMD. After adjustment for age, non-African-American race, methadone use, and LBM, HIV therapy was not associated with lower WBMD (–0.002, *p* = .89).

**Table 2:** Potential Determinants of Whole-Body BMD: Analysis by Gender

	Male		Female	
		
Variable	Univariate β estimate	Multivariate^a^ β estimate	Univariate β estimate	Multivariate^b^ β estimate
Lean body mass (kg)	0.005***	0.006***	0.007***	0.006***
Age (per increase of 5 years)	0.003	0.002	−0.03**	−0.02***
Race (non-African American/African American)	**−**0.04	0.04	−0.18***	−0.14***
Methadone	**−**0.03	NS	−0.05*	−0.05***
HIV^c^	0.02	NS	**−**0.04	**−**0.03
Smoking^d^	0.02	NS	**−**0.04	NS
Alcohol^e^		NS		NS
0 (no use)	Ref		Ref	
1 (<3 drinks/day)	0.03		0.05*	
2 (≥3 drinks/day)	**−**0.007		0.01	
Drug use^f^		NS		NS
0 (no use)	Ref		Ref	
1 (<3 times/week)	0.005		0.01	
2 (≥3 times/week)	**−**0.02		0.05	
Hypogonadal^g^/menopausal^h^	**−**0.01	NS	−0.1***	NS
Hepatitis C^i^	0.005	NS	**−**0.02	NS
Fat body mass (kg)	<0.01	NS	< 0.01*	NS
BMI (kg/m^2^)	0.007**	NS	0.005**	(Not included)
DHEA (tertiles)^j^	NS		NS	
1	Ref		Ref	
2	**−**0.04		0.05	
3	0.0004		0.07**	
Estradiol (tertiles)^j^		NS		NS
1	Ref		Ref	
2	**−**0.003		0.04	
3	0.02		0.08**	
IL-6 (tertiles)^j^		NS	NS	
1	Ref		Ref	
2	**−**0.006		**−**0.01	
3	**−**0.004		**−**0.02	
CRP (tertiles)^j^		NS		NS
1	Ref		Ref	
2	**−**0.03		0.03	
3	**−**0.007		0.03	
TNF-α (tertiles)^j^		NS		NS
1	Ref		Ref	
2	**−**0.03		**−**0.03	
3	0.006		**−**0.03	

NS = nonsignificant.

*Note:* Items in **boldface** denote significant *p* values:

* *p* < .05.

** *p* < .01.

*** *p* < .0001.

a Final model *R*^2^ value = 0.12, *R*^2^ adjusted = 0.1, *p* < **.0001**, *n* = 186.

b Final model *R*^2^ value = 0.38, *R*^2^ adjusted = 0.36, *p* **< .0001**, *n* = 150.

c Self-report, HIV^+^ by testing, or history of past or present use of antiretroviral therapy.

d Determined by answering yes to having smoked cigarettes in the past 6 months (irrespective of quantity).

e Equivalent of 1 drink is 8 fluid ounces of beer or wine, 4 fluid ounces of liquor or malt, and 1 fluid ounce of vodka.

f Includes marijuana, crack/freebase, cocaine (snorting, injection), speedball (injection), heroin (smoking, snorting, injection), and street methadone in the past 6 months.

g Defined as having free testosterone level < 52 pg/mL or currently on testosterone-replacement therapy.

h Defined as having an FSH level > 50 mIU/mL, age ≥ 51 years, or an FSH level >30 and ≤50 mIU/mL and answered yes to having gone through menopause.

i Self-report.

j Obtained by plotting continuous lab values, 0 representing the lowest tertile, 1 the middle tertile, and 2 the highest tertile.

**Table 3:** Bone Mineral Density Measures

Site Measure	Male	Female	Total
Whole-body data (*n* = 338)	(*n* = 187)	(*n* = 151)	
Total BMD mean (g/cm^2^) (SD)	**1.27 (0.13)**	**1.19 (0.13)**	1.23 (0.14)
Site-specific data (*n* = 132)	(*n* = 83)	(*n* = 49)	
Total hip			
BMD mean (g/cm^2^) (SD)	1.02 (0.15)	0.98 (0.15)	1 (0.15)
Proportion with reduced BMD^a^	**42 (50.6%)**	**12 (24.5%)**	54 (41%)
Proportion with osteopenia^b^	**40 (48.2%)**	**11 (22.4%)**	51 (38.6%)
Proportion with osteoporosis^c^	2 (2.4%)	1 (2%)	3 (2.3%)
Hip-femoral neck			
BMD mean (g/cm^2^) (SD)	0.94 (0.15)	0.90 (0.15)	0.93 (0.15)
Proportion with reduced BMD^a^	**39 (46.9%)**	**12 (24.5%)**	51 (38.6%)
Proportion with osteopenia^b^	**37 (44.6%)**	**11 (22.4%)**	48 (36.4%)
Proportion with osteoporosis^c^	2 (2.4%)	1 (2%)	3 (2.3%)
Lumbar spine			
BMD mean (g/cm^2^) (SD)	1.04 (0.15)	1.04 (0.15)	1.04 (0.15)
Proportion with reduced BMD^a^	54 (65%)	25 (51%)	79 (60%)
Proportion with osteopenia^b^	39 (47%)	21 (42.9%)	60 (45.5%)
Proportion with osteoporosis^c^	**23 (27.7%)**	**5 (10.2%)**	28 (21.2%)
Reduced BMD any site^d^	**63 (75.9%)**	**28 (57%)**	91 (68.9%)
Osteopenia any site^d^	46 (55.4%)	23 (46.9%)	69 (52.3%)
Osteoporosis any site^d^	**24 (28.9%)**	**5 (10.2%)**	29 (22%)

*Note:* Items in **boldface** denote statistically significant difference (*p* < .05) between males and females.

a Reduced BMD defined as having a *T*-score < –1.

b Osteopenia defined as having –2.5 < *T*-score < –1.

c Osteoporosis defined as having a *Z*-score ≤ –2 for pre-menopausal women and men < 50 years of age and *T*-score ≤ –2.5 for postmenopausal women and men ≥ 50 years of age.

d Total hip, hip femoral neck, or lumbar spine.

### Demographic and BMD characteristics of the subpopulation with site-specific DXA

**Fig. 1:**
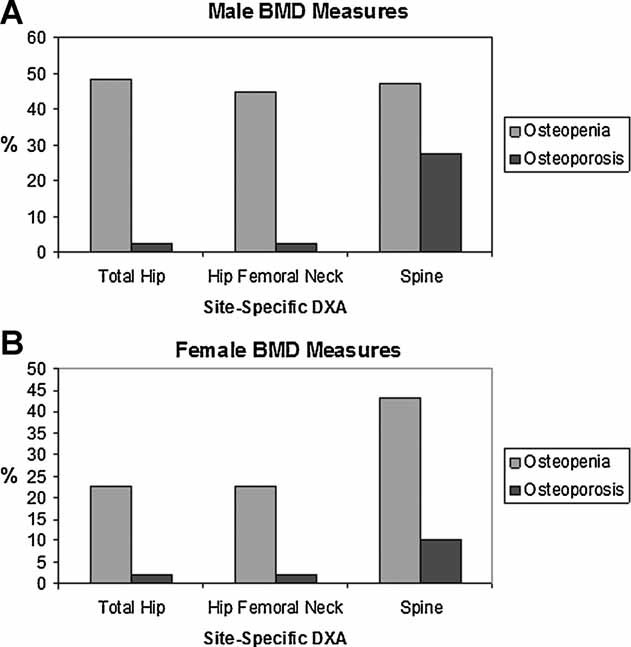
(*A*) Male BMD measures. (*B*) Female BMD measures

Of the 338 subjects, 132 subjects received site-specific DXA scans of the spine and hip. Demographic characteristics of this group were similar to those of the larger cohort (Table 1), with similar distribution among men and women (not shown). Men had lower BMI values than women (24.4 versus 26.2 kg/m^2^, respectively), lower fat mass, but higher LBM (59.3 versus 46 kg, respectively) as well as higher WMBD (1.26 versus 1.20 g/cm^2^, respectively), all with statistically significant differences between men and women (*p* < .05). Regression analysis revealed strong correlation between WMBD and spine BMD (*r* = 0.57, *p* < .0001), between WMBD and total-hip BMD (*r* = 0.53, *p* < .0001), and between WBMD and hip femoral neck BMD (*r* = 0.46, *p* < .0001). Reduced BMD was present in 69% of the population, with twice as many men having osteoporosis of the hip region (total or femoral neck) compared with women. Osteoporosis, as defined by the NOF, was identified in 22% of subjects (24 men and 5 women), with the majority of cases (90%) attributable to osteoporosis of the lumbar spine (Table 3, Fig. 1*A, B*).

### Risk factors associated with osteoporosis

The relation between osteoporosis and different independent variables is shown in Table 4. Men were nearly four times more likely to have osteoporosis than women [odds ratio (OR) = 3.6, 95% confidence interval (CI) 1.3–10]. Increasing LBM was the only other variable associated with lower risk of osteoporosis (OR = 0.84, 95% CI 0.76–0.94) in univariable analysis (Table 4). Similar results were obtained when BMI replaced lean and fat mass.

**Table 4:** Potential Determinants of BMD by Site-Specific DEXA: Odds Ratio (95% CI) for having Osteoporosis^a^

Variable	Univariate analysis	Multivariate^b^ analysis
Gender (male/female)	3.58 (1.27–10.12)*	22.9 (5.01–105.5)***
Lean body mass^c^ (kg)	0.97 (0.93–1.01)	0.88 (0.825–0.945)**
Age (per increase of 5 years)	0.84 (0.65–1.08)	0.86 (0.64−1.14)
Race (non-African American/African American)	3.96 (0.93–16.94)	3.32 (0.71−15.4)
Smoking^d^	0.7 (0.26–1.89)	NS
Alcohol^e^		NS
0 (no use)	Ref	
1 (<3 drinks/day)	1.07 (0.43–2.67)	
2 (≥ 3 drinks/day)	1.82 (0.52–6.32)	
Drug use^f^		NS
0 (no use)	Ref	
1 (<3 times/week)	0.74 (0.21–2.58)	
2 (>3 times/week)	1.26 (0.4–3.97)	
Hypogonadal^g^/menopausal^h^	1.01 (0.4–2.53)	NS
Hepatitis C^i^	1.22 (0.53–2.84)	NS
HIV^j^	1.77 (0.76–4.12)	NS
Fat body mass (kg)	**0.99 (0.99–0.99)**	NS
BMI (kg/m^2^)	0.84 (0.76–0.94)**	NS
Methadone	1.52 (0.61–3.8)	NS
DHEA (tertiles)^k^		NS
1	Ref	
2	2.43 (0.85–6.95)	
3	1.15 (0.39–3.42)	
Estradiol (tertiles)^k^		NS
1	Ref	
2	1.35 (0.47–3.82)	
3	0.55 (0.17–1.77)	
IL-6 (tertiles)^k^		NS
1	Ref	
2	0.77 (0.25–2.35)	
3	0.78 (0.23–2.63)	
CRP (tertiles)^k^		NS
1	Ref	
2	0.49 (0.15–1.56)	
3	0.32 (0.1–1.06)	
TNF-α (tertiles)^k^		NS
1	Ref	
2	1.08 (0.35–3.27)	
3	0.46 (0.13–1.6)	
Vitamin D deficient^l^		NS
0	Ref	
1	0.69 (0.29–1.59)	
Secondary hyperparathyroidism^m^		NS
0	Ref	
1	1.15 (0.41–2.97)	

NS = nonsignificant.

*Note:* Items in **boldface** denote significant *p* values

* *p* < .05.

** *p* < .01.

*** *p* < .0001.

a Osteoporosis defined as having a *T*-score ≤ –2.5 for postmenopausal women and men aged ≥ 50 years of age or a *Z*-score ≤ –2 for premenopausal women and men < 50 years of age. *T*-score/*Z*-score measured at the lumbar spine, total hip, or hip femoral neck (lowest value taken).

b Final model, *R*^2^ = 0.193; max-rescaled *R*^2^ = 0.297, *p* = **.0001**, *n* = 132.

c In kilograms, measured by whole-body DXA scan.

d Determined by answering yes to having smoked cigarettes in the past 6 months (without account to quantity).

e Equivalent of 1 drink is 8 fluid ounces of beer or wine, 4 fluid ounces of liquor or malt, and 1 fluid ounce of vodka.

f Includes marijuana, crack/freebase, cocaine (snorting, injection), speedball (injection), heroin (smoking, snorting, injection), and street methadone in the past 6 months.

g Defined as having a free testosterone level < 52 pg/mL or currently on testosterone-replacement therapy

h Defined as having an FSH level > 50 mIU/mL, age ≥ 51 years, or having FSH > 30 or ≤ 50 mIU/mL and answered yes to having gone through menopause.

i Self-report.

j Self-report, HIV^+^ by testing, or history of past or present use of antiretroviral therapy.

k Obtained by plotting continuous lab values: 0 = the lowest tertile, 1 = the middle tertile, and 2 = the highest tertile.

l Deficiency defined as having 25(OH)D level ≤ 15 ng/mL.

m Defined as having an iPTH level > 65 pg/mL.

Of the 126 subjects with site-specific DXA and biochemical measurements of 25(OH)D and iPTH, 124 (98%) had 25(OH)D level < 32 ng/mL, 72% had 25(OH)D level ≤ 20 ng/mL, and 48% had 25(OH)D level ≤ 15 ng/mL. Secondary hyperparathyroidism (iPTH > 65 pg/mL) was present in 22% of subjects. 25(OH)D was inversely related to iPTH (*r* = 0.08, *p* = .001). However, the prevalence of secondary hyperparathyroidism was similar in those with and without vitamin D deficiency (28% versus 17%, *p* = .14). Osteoporosis was not associated with vitamin D deficiency or secondary hyperparathyroidism (Table 4). Site-specific *Z*-scores were similar regardless of the presence of vitamin D deficiency and/or secondary hyperparathyroidism (Table 5).

**Table 5:** Site-Specific *Z*-score (Mean, SD) by Vitamin D, PTH Status

	Normal PTH		High PTH (>65 pg/mL)	
		
Site	Low vitamin D^a^ (*n* = 44)	Normal vitamin D (*n* = 54)	Low vitamin D^a^ (*n* = 17)	Normal vitamin D (*n* = 11)
Total hip	−0.35 (1.0)	−0.22 (0.90)	−0.19 (0.9)	−0.47 (1.0)
Femoral neck	−0.04 (1.1)	0.04 (0.99)	0.24 (1.1)	−0.23 (0.8)
Lumbar spine	−0.81 (1.5)	−0.99 (1.3)	−0.92 (1.3)	−1.36 (1.4)

Values presented as mean (SD).

a Defined as 25(OH)D ≤ 15 ng/mL.

## Discussion

In this predominately middle-aged, low-income, African-American, inner-city population in Baltimore, MD, we found a very high prevalence of osteoporosis, particularly at the lumbar spine. BMD in the osteoporotic range was more common in men than in women. Among men, only lower LBM was a significant positive determinant of lower WBMD, whereas in women, multiple factors were associated with lower WBMD, including age, white race, methadone use, and lower LBM.

The prevalence of osteoporosis was higher than expected in this population, which was 94% African American. Race plays an important role in BMD, where African Americans attain higher peak mass than whites, Hispanics, and Asians during puberty.(18,19) In population-based studies, African Americans have 13% to 18% higher hip BMD measures(20–24) and 2% to 12% higher spine BMD measures(20–25) than whites. The National Health and Nutrition Examination Survey III (NHANES III) data, consisting of 7116 subjects, similarly reported 12.3% higher femoral neck BMD measures in African-American compared with white men.(26,27) Compared with African Americans in a random sample from Boston (mean age 48 years), where the majority had good health, our African-American men had similar WBMD and femoral neck BMD measures, but our men had 6.4% lower total-hip and 6.3% lower spine BMD measures.(20) Other populations of African Americans also have shown high rates of osteoporosis. In a population of African-American male veterans (mean age 64 years, BMI 29 kg/m^2^), the prevalence of osteopenia (39%) and osteoporosis (7%) was unexpectedly high in a relatively low-risk population.(28) The contributing factors associated with low BMD in this cohort were not described. Together with our findings, these observations suggest that clinicians should be aware that African-American race is not necessarily a protective factor for low BMD. While we cannot conclude that African-American race is associated with lower BMD because our cohort was overwhelmingly African American and no similar cohort of white subjects is available for comparison, our data suggest that more aggressive bone density screening may be appropriate in some African-American populations.

Another major finding in our study was the higher prevalence of osteoporosis in men than in women. Rates of BMD lower in men than in women have been described among groups with depression(29) and in subjects on methadone maintenance, where in one study the prevalence of osteoporosis was higher in men than in women (61% versus 20%).(9) Plausible explanations for such high rates among men included secondary causes of osteoporosis such as reduced physical activity and diet. In our study, we investigated some secondary causes such as hypogonadism, vitamin D deficiency, and alcohol and tobacco abuse; however, none was significantly related to lower BMD.

The prevalence of vitamin D deficiency was high in our DXA subpopulation [48% with 25(OH)D < 15 ng/mL], a finding that is common among African Americans.(30–32) However, neither vitamin D deficiency nor secondary hyperparathyroidism could account for the high prevalence of osteoporosis in our population. It has been suggested that the adverse effects of vitamin D deficiency on bone in African Americans are less pronounced than in whites, possibly owing to relative PTH resistance.(33) This hypothesis requires further investigation because it has clear implications for the definition of vitamin D deficiency in African-American populations.

Most of the osteoporosis in our cohort (>90%) was found in the spine. The spine consists mainly of trabecular bone, which is highly metabolically active and is characterized by increased bone remodeling. In young men, loss of trabecular bone has been related to low physical activity.(34) Lower-spine BMD can have important clinical consequences. In the Osteoporotic Fractures in Men (MrOS) study, a prospective community study of 5995 men aged 65 years and older, a significant association was observed between lumbar spine BMD and vertebral fractures and a weaker but significant association between lumbar spine BMD and risk of hip fracture.(35,36) In contrast to the population of MrOs, which was predominantly white (90%), our population was 94% African American. It is unknown whether the low spine BMD observed in our cohort is predictive of higher rates of clinical vertebral and nonvertebral fracture because historical data regarding incidence fractures after DXA assessment were not captured.

The risk factors for lower BMD in our cohort differed between men and women. In both men and women, LBM was an important correlate of BMD. However, among women, other factors were associated with lower BMD, including age, non-African-American race, methadone use, and HIV status. Other studies have shown differences in risk factors among women and men. The Framingham Osteoporosis Study, which examined risk factors for longitudinal bone loss, found only weight to be related to lower BMD in men, whereas weight, alcohol intake, and estrogen therapy constituted risk factors in women.(37) Consistent risk factors for low BMD in a systematic review of healthy men aged 50 years or older included advanced age, smoking, and low weight.(38) Since our population was enriched by injection drug users having multiple risk factors that are different from the populations studied in the literature, other risk factors might be present that merit further investigation as low BMD determinants among men in this population.

Consistent with the literature, BMI was a significant determinant of BMD in both men and women.(39,40) When evaluating the components of BMI that contribute to the relation with BMD, our analysis showed that LBM rather than fat mass predicted BMD in both women and men compared with some studies that found LBM in men but fat mass in women to be BMI mediators.(41–43) Despite the high prevalence of many osteoporosis risk factors, surprisingly, we found no association between heavy alcohol, drug, and tobacco use; hepatitis C infection; hypogonadism; and inflammatory markers and lower BMD.

### Implications and Conclusion

Osteoporosis of the spine was strikingly high in this cohort of inner-city, high-risk African-American males. Although the causes of this finding in our population are unclear, the high prevalence of osteoporosis in this young population could indicate even higher rates in similar older subjects. There is evidence of suboptimal recognition and diagnosis of osteoporosis among African Americans and in men,(44–46) and such disparities in detection can be more pronounced in underserved populations such as ours. Current osteoporosis guidelines suggest DXA screening in postmenopausal women and men aged 50 to 70 years based on clinical risk factors. Given the high burden of osteoporosis in this population, screening for osteoporosis could be warranted in this high-risk subpopulation at a younger age (50 years). It is uncertain that such high rates of osteoporosis could translate to increased fracture rate in the future, but further studies are needed to better understand and assess the implications.
